# Video cases as tricksters, in medical students´ transition to psychiatric clerkship. A liminal perspective

**DOI:** 10.15694/mep.2021.000095.1

**Published:** 2021-04-20

**Authors:** Kamilla Pedersen, Anne Mette Moercke, Charlotte Paltved, Ole Mors, Charlotte Ringsted

**Affiliations:** 1 Aarhus University; 2 Copenhagen Academy for Medical Education and Simulation; 3 Corporate HR; 4 Psychosis Research Unit; 5 Centre for Health Sciences Education

**Keywords:** Clerkship, Liminality, Medical students, Psychiatry, Video Cases, Patient-centred Learning, Educational Technology, Professional Behavior, Educational Anthropology

## Abstract

This article was migrated. The article was marked as recommended.

Objective:

This study introduced a lens of liminal theory, drawn from anthropological classical ritual theory, to explore how a preparatory teaching format using video casesinfluenced medical students’ patient approaches in their subsequent psychiatric clerkship. The video cases portrayed simulated patient-doctor encounters in diagnostic interview situations and were hypothesized to function as a liminal trickster.

Methods:

The study applied a qualitative explorative design using individual rich picture interviews. We asked the students to draw their experiences, which we investigated using a semi-structured interview guide designed to capture and unfold the students’ perspectives. We explored how students navigated insights from the preparatory teaching in their clerkship using liminal theory concepts in a mixed inductive and deductive thematic analysis.

Results:

The results from 8 rich picture interviews demonstrated that students’ ability to navigate insight gained from the video cases in their clerkship varied according to their roles in the clinical diagnostic interview situations. Students having active roles in the diagnostic interview situation adopted a patient-centred focus demonstrating empathic engagement and self-reflexivity related to their learning experiences with the video cases. Students with passive roles described a focus on how to adopt an appropriate appearance and copied the behaviour of the simulated doctors in the video cases.

Conclusion:

The liminal ritual theory perspective to explore the influence of preparatory teaching was useful for demonstrating how video cases could affect students’ patient-centred learning. Without guidance and active roles in clerkship, medical students’ learning experiences may lead to a prolonged liminal phase and may not capitalise on the potentially positive effects of the preparatory teaching. Liminal theory may further inform our understanding of students’ learning considering patient cases in educational technology arrangements as tricksters.

## Introduction

Communication in psychiatry represents a challenge for medical students, who are often anxious about how to meet psychiatric patients ( Gask, Sholevar, Bizamcer and Garbely 2009; Lewis, 2013). Preparatory teaching of medical students on how to deal with psychiatric patients is mostly mediated through the format of a text-based patient case. However, the complexities present when meeting and communicating with psychiatric patients in the clinic are difficult to depict in text-based material ( Graf *et al*., 2014), and presenting patient cases in the format of a text has met critique for taking the doctor’s viewpoint and for being divorced from the patient’s perspective. Patient cases as text are therefore considered less optimal in teaching communication skills because they lack active student engagement and the patient’s perspective and convey depersonalized and decontextualized views ( Kenny and Beagan, 2004; Pomata, 2014). Another way to prepare medical students to meet, engage and communicate with patients, before entering their psychiatric clerkships, is by using video cases portraying the patient-doctor encounter ( DeLeng *et al*., 2007; Magliano., *et al.,* 2014). However, we know little about how video cases influence students’ learning in their subsequent engagement with psychiatric patients in their clerkship ( Courteille, 2008; Levinson and Lesser, 2010; Gask, Coskun and Baron 2011).

In medical education, transition to clerkship is regarded as stressful, as students must cope with new responsibilities and expectancies ( Belitsky and Kennedy, 1995; Tazakori *et al*. 2008). The transition to the psychiatric clerkship has unique challenges as the psychiatric diagnostic process lacks the supporting biological variables that students are familiar with in somatic specialities. Hence, the diagnostic interview is central to psychiatric practice ( Belitsky and Kennedy, 1995; Sartorius *et al*., 2010). Stigma regarding psychiatry and psychiatric patients is known to add to anxiety and insecurity in medical students, negatively influencing their communication skills and subsequent diagnostic accuracy ( Kassam *et al.* 2011; Lyons, 2014; Magliano *et al.* 2014). Consequently, medical schools need to prepare medical students before their clerkships for the particular skills and emotional challenges of communicating with psychiatric patients ( Sartorius *et al.* 2010; Levinson and Epstein, 2010). As a part of this preparation, video cases may serve an important role. Video cases can portray clinical practice and intensify students’ learning experience and reflection ( Kerres and Witt, 2003; Kenny and Beagan, 2004; Chan *et al.* 2010). Two previous studies suggests that using video cases in preparatory teaching foster patient-centred perspectives better than text-based patient cases prior to meeting psychiatric patients ( Pedersen *et al.*, 2018; Pedersen *et al*. 2019). In this study, we sought to widen our understanding of how a preparatory teaching format using video cases influences medical students’ patient approaches during their experiences in psychiatric clerkship.

### Theoretical framework

The theoretical framework of this study is liminal theory ( Adorno, Cronley and Smith, 2013). The concept of liminality stems from classical anthropological studies of rituals. Liminal theory operates with a ‘ritual elder’ who foregrounds the ‘in-betweenness’ status of the participants undergoing transition ( Turner, 1967). The ritual elder passes on the traditions to the youngers through storytelling and by the use of the liminal ‘trickster’ which appears in the myths and rituals of many different cultures where learners cross thresholds from one status to another ( Turner, 1966; Hyde, 2008). The trickster refers to an individual or a figure in storytelling who challenges the existing socio-cultural order by questioning and drawing attention to particular assumptions and practices ( Spandler, 2009). The ritual elder uses tricksters as a tool in his or her storytelling to trigger disruptive emotions of confusion and contradict prior beliefs and knowledge, subsequently catalysing reflection to guide the learners beyond what they know, leading to new insights ( Turner, 1974; Davis and Weeden, 2009; Priyadharshini, 2012). Ultimately, learners are ‘incorporated’ or ‘reborn’ with their newly achieved insight to a new social status and new roles in the post-liminal setting ( Blows *et al*., 2012).

Teaching has been considered as having ritual-like features with similar structures that move students from one status to another ( Cook-Sather and Zanny, 2011). The liminal concepts, including liminal space, ritual elder, and tricksters, could align with preparatory teaching in medical education, as the medical student moves through a rite of passage from the preparatory teaching setting to the practice setting in the clerkship ( Belitsky and Kennedy, 1995; Tazakori *et al*., 2008). In this analogy, a lecturer exposes students to storytelling and tricksters during teaching to help the students navigate their transition to practice. Teaching formats such as video cases portraying simulated psychiatric patients and simulated doctors could be considered tricksters, as they aim to challenge and disrupt beliefs and preconceptions of psychiatry and give the students a new professional insight into clinical psychiatry. Following the rite of passage analogy, clerkship experiences could be regarded as a post-liminal phase, where students integrate and adapt to the hospital culture with their newly acquired insight and skills ( Davys and Beddoe 2009; Jones and Shapiro 2012; Teunissen and Westerman 2011; Janssen and Walker 2008). Thus, liminal theory as a lens could offer a dynamic and situated understanding of transition between preparatory teaching and clerkship experience.

From this framework, our research question was:

“How does a preparatory teaching format, using video cases portraying the diagnostic interview with simulated patients and doctors, influence medical students’ learning in psychiatric clerkship?”

## Methods

### Context and participants

The context of the study was an existing 4-week clinical course in psychiatry for fourth-year medical students at Aarhus University. The 4-week course included large-group teaching with preparatory lectures and subsequent clinical placements. Three 2-hour lectures in diagnostic interviews prepared students for the diagnostic interview with psychiatric patients.

For the purposes of the study, we designed a video-based teaching format using three patient cases, covering mania, psychosis, and depression. To avoid cognitive overload, the video cases did not exceed 20 minutes. They were structured in sequences not exceeding 2 minutes. The cases aimed to portray common interpersonal encounters between patients and clinicians, rather than uncommon and potentially dramatic situations. For example, the video cases did not portray violent and aggressive patients or threatening situations. Such situations may occur in clinical practice, but they are not representative of everyday practice encounters. These video cases were designed to function as tricksters. The theme of the three video cases was the diagnostic interview portrayed as a dialogue between a patient and a doctor, both played by actors. The three video cases were applied to an interactive e-learning format. Each video case contained a starting sequence followed by 8-12 built-in open questions on the observed and which patient-perspectives to prioritise and explore further to identify symptoms. One senior lecturer systematically used the video cases during one of the 2-hour preparatory lectures to elaborate on clinical practice by using the built-in questions. The lecturer initiated reflection and encouraged dynamic discussions among the large group of students.

102 medical students who had followed the preparatory lectures in spring 2016 were allocated by faculty administration to their clerkship at one of nine psychiatric departments located in six hospitals in the Central Denmark Region.

By e-mail, we invited students in the final week of their clerkship to participate in an explorative interview. Students’ e-mail and rotation information was provided by the faculty administration. We excluded students with clerkship placement in child, adolescent, and forensic psychiatry, as these departments did not let students practise diagnostic interviews.

### Interviews

Our study was an explorative qualitative study using rich pictures as a visual approach to provide an understanding of the medical students’ experiences ( Monk and Howard, 1998; Spickard, 2016). Asking participants to draw their experiences in combination with interviews has the ability to provide a “rich picture” that can unfold tacit knowledge, perceptions, and emotions of complex situations in professional practice ( Cristancho, 2015).

Three researchers, CP, AM and KP, made a semi-structured interview guide in an iterative process ( Kvale, 1996). The interview guide included questions aiming to explore students’ drawings with a focus on their experiences, emotions, and conflicts in the contextual situation ( Cristancho *et al.,* 2014; Liamputtong, 2011; Kvale, 1996). The interview guide was pilot-tested with two fourth-year medical students, reviewed by two associate professors in health science education with qualitative interview experience, and refined accordingly.

The interviews were scheduled to last for 2 hours to enable the participant to feel comfortable in engaging in a dynamic discussion ( Liamputtong, 2011). One interviewer (KP) and one observer were present. All interviews followed the same structure: First, the study was explained to the medical student to obtain verbal and written consent. Second, they were introduced to the visual approach ( Cristancho *et al.,* 2014) and instructed to draw on an A3 paper sheet in silence for 30 minutes. The written instruction was:

“Reflect upon and draw a situation from the clerkship where the preparatory video-based teaching format helped you navigate meeting and communicating with a psychiatric patient. Try to illustrate everything you perceive to be part of the situation, including ideas, people, and connections. You can also draw representations of more subjective aspects, such as figures with human characteristics, feelings, conflicts, and prejudices.”

Finally, the interviewer used the semi-structured interview guide to invite open and descriptive reflections relating to the student’s drawing. All interviews were recorded and transcribed verbatim and the drawings scanned.

### Data Analysis

We used a thematic analysis inspired by Braun and Clarke to search for patterns of meaning in relation to the medical students’ liminal experiences ( Braun and Clarke, 2006). Initial codes suggested by KP and AM were discussed with the author group. This was an iterative process, as recommended by Kvale with constant comparison and identification of codes and patterns in the entire dataset ( Kvale, 1996). We adapted and refined codes as ideas and themes emerged. As we explored patterns relating to students’ experiences and perceptions, themes of liminal theory concepts were identified. Authors reached consensus on all themes with the identification and comparison of occurring codes. We reached thematic saturation within eight interviews.

## Results/Analysis

Two main findings emerged from the analysis. First, students’ reported insight gained from the video-case tricksters depended on whether the students had an active or passive role in clinical interview situations. Second, the presence of a ritual elder was paramount to students’ ability to reflect upon and gain professional insight from trickster experiences in the preparatory teaching as well as the clerkship setting.

Using insight from the video case tricksters to navigate practice

Students describing an active role in the diagnostic interview situations adopted a patient-centred focus, explored patient perspectives, demonstrated empathic engagement, and reflected on their initial impressions and emotions with references to insight drawn from the video cases.

“She [patient in clerkship] made me feel moved - as I felt when confronted with the patient in the lecture [the video case]. She [patient in clerkship] has a family who cares for her, but still she does not want to be a burden. Therefore, I asked her about her family, so we talked about this. I think that both patients had tendencies to isolate themselves because they did not want to be a burden to their families.”

**Figure 1.**
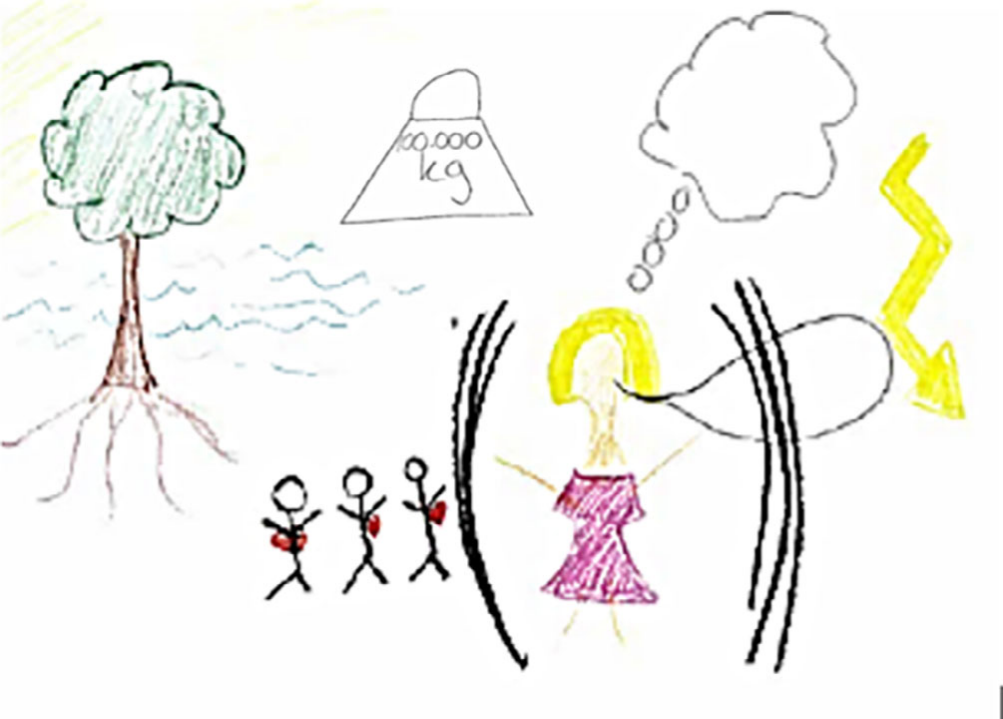
Student illustration with hearts on small people figures as an allegory for a caring family

The shield around the patient was an allegory conveying a tendency to isolation, and the weight was an allegory for feeling like a burden.

In contrast, the content from the preparatory lecture and video cases was emphasised differently if the student had a passive observer role, described as sitting next to doctors in the diagnostic interview situation. Students with passive roles demonstrated relatively simple reflections about their own appearance. They pictured how the doctor portrayed in the video cases had looked and acted and described how they copied the behaviour of the simulated doctors during the clinical diagnostic interview situation.

“You have to be able to not react in an awkward way when the patients tell you things that are very dramatic. What I thought was good about the video cases was that you did not only see the patients’, but also the doctors’, facial expressions. It gave me some tools to help me act above the table - appearing very open. However, I curled up my legs under the table hoping none of them [doctor and patient] saw how I trembled.”

**Figure 2.**
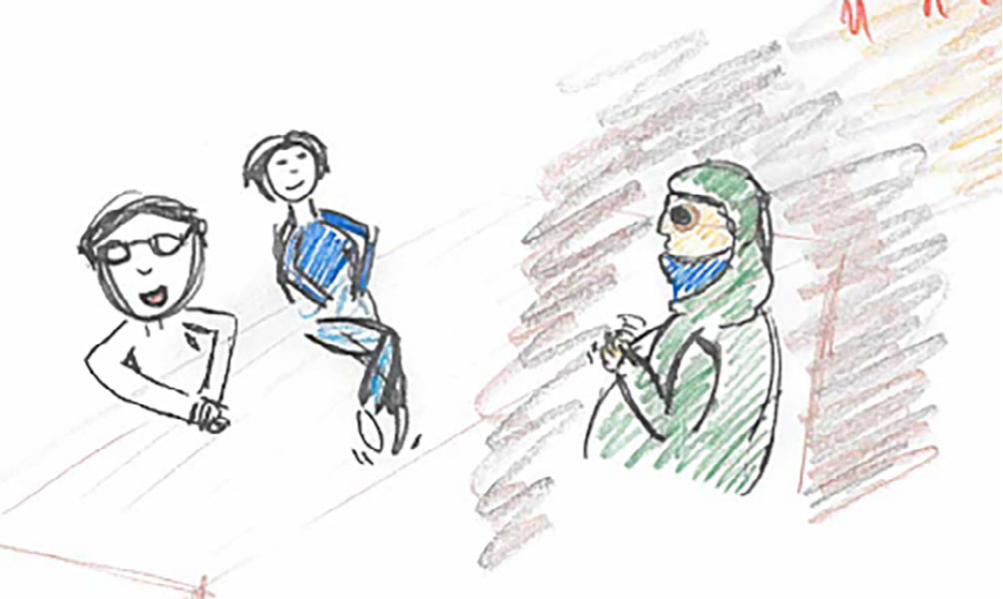
Student illustration with shadow around the patient as an allegory for the patients suffering

The students’ legs are curled up and trembling under the table; the student poses with a smiling face and eyes focused at the patient above the table, illustrating mimicking the doctor in the video case. The patient is depicted with dark eyes as an allegory for having dark thoughts.

The critical role of a ritual elder for learning from video-case tricksters and clerkship tricksters

The senior lecturer acted as a ritual elder in the preparatory teaching and guided students to new insights following the emotive disruptions caused by the video-case tricksters.

“The additional perspectives and explanations of the lecturer gave some ‘aha’ experiences on why the doctor in the video had asked some questions in such a direct manner [suicide] which I would not have perceived on my own. Perhaps one disagreed about the approach in the video, but addressing and discussing such issues with the lecturer in class made you understand.”

Using the liminal perspective as a lens for understanding the students’ clerkship experiences, we found that their ‘liminal space’ experience of being betwixt-and-between was not limited to the preparatory lectures, but extended to the students’ transition to their clerkship. Entering the clerkship, various experiences could be considered as having trickster-like abilities in addition to the video cases. As the students were confronted with the variety of trickster-like experiences in their clerkship, an additional need for guidance and support to understand and grasp these experiences emerged. Again, we found that students differed in how they used the video-case trickster to navigate the transition to clerkship. If students encountered hospital staff, who functioned in the role of a “ritual elder”, students demonstrated an explorative and patient-centred understanding similar to the reflective process they had experienced with the video cases.

“...I told the resident doctor that I am a little nervous about entering the psychiatric ward. He asked what caused this and I told him that I thought it must be prejudice emerging, because you only hear about the bad things in the media. He then told me that their focus was to make me feel safe and learn that psychiatry is not dangerous. I felt relieved to recognise they [hospital staff] also care about you. Experiencing the video with the patient in the lectures and being encouraged by the hospital staff to enjoy, listen and figure out what is going on and accept that not everything can be fixed, the psychiatric patients, I found, are diverse and function in society one way or another, just like us.”

In contrast, if the students lacked encouragement, support and guidance from the hospital staff, the psychiatry clerkship was described as emotionally disruptive and the video cases were perceived as less relevant.

“We [students] are just like flies, looking for a doctor to cling onto for a while. Nobody is concerned about your [medical students’] thoughts, needs and feelings. The clerkship feels less authentic as you are not given the opportunity to practise. We are not encouraged or given access to patients. Why, I do not understand. In less than a year [as post-graduate doctors], we have to do it anyway with no lifelines. I think - then I could use such video cases when I am in the real world. Not in the clerkship.”

Lacking a “ritual elder”, students described their feelings of anxiety when meeting admitted patients displaying an intrusive and aggressive behaviour. Students felt uncomfortable and lacked the ability to handle such situations when they perceived no or insufficient guidance from resident staff. The students described how they had coped with the stressful situations by considering how to place one-self with reference to the video cases, in case a potentially dangerous situation should occur.

“It was the first thing I drew - the escape route as the door behind the patient. I have mainly been to a psychosis ward. Some have delusions - which I have tried to illustrate with a chip - or something they believe they have had implanted. So you have to always remember to be oriented in how you could exit a room if something should happen, and having the table between you and the patient, like in the video.”

**Figure 3.**
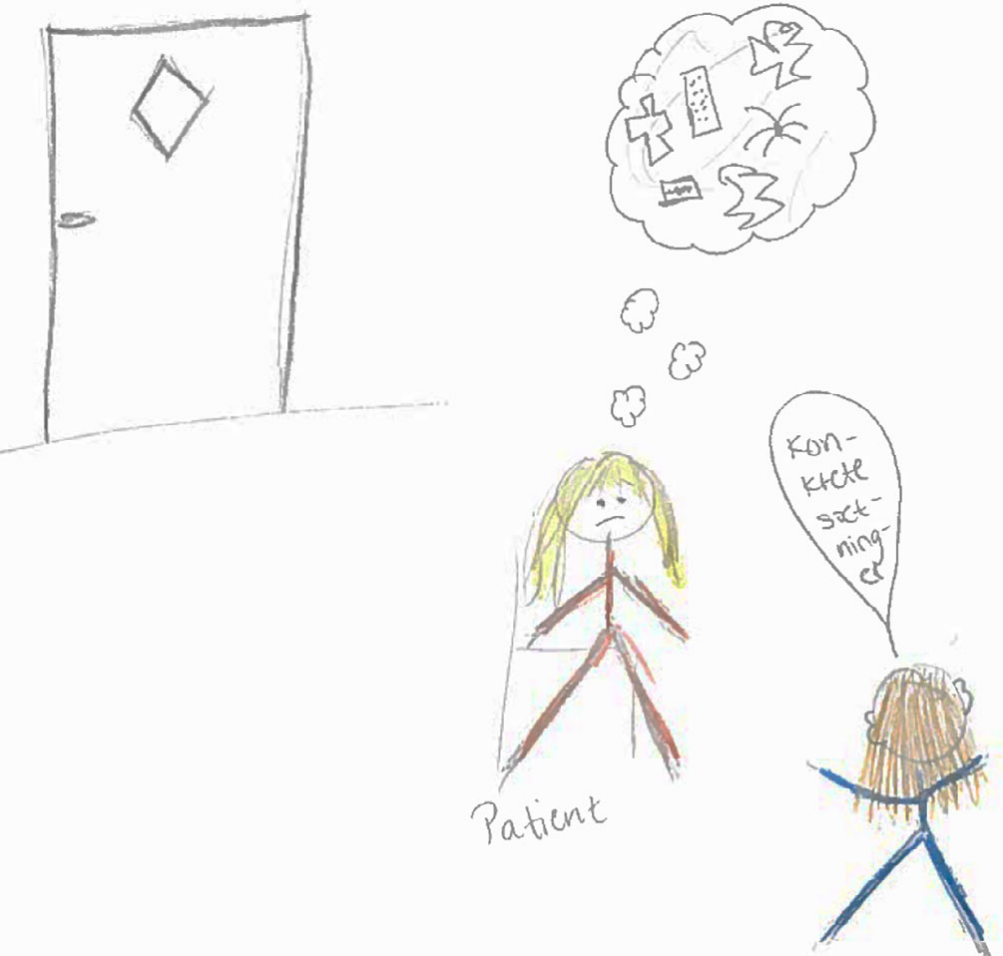
Student illustration of awareness of escape routes.

The patient in the drawing, illustrated with psychotic delusions and agitated behavior, stands between the medical student and the door, demonstrating a potentially dangerous situation as the medical student cannot flee through the door if his/her communication fails to calm the patient.

## Discussion

This study demonstrates how medical students use insight from video cases in a preparatory teaching format to transition to and navigate their clinical practice. The influence of the video cases depended on whether students had an active or a passive role in practising the diagnostic interview and whether the students received encouragement and support in their clerkship from a ritual elder. The role of a ritual elder was significant for learning from the preparatory teaching and the clerkship alike.

### Active and passive clerkship roles influence on transition initiated in preparatory teaching

Successful integration of the new insight seems to depend on students’ ability to enact the active role of a doctor portrayed in the video cases. Students having active roles demonstrated self-reflexivity when describing how they used insight gained from discussing the video cases. Successful transition to the post-liminal phase is characterised by such self-reflexivity and deliberate integration of insight achieved within the liminal space ( Davys and Beddoe, 2009). In contrast, students having predominantly passive roles focused on their own appearance and adopted a form of mimicry of the doctor’s appearance in the video cases. In liminal rituals, the weak participants mimic superiors as a defence mechanism against objects of their fear ( Turner, 1995). Mimicry in students has previously been identified as a method to cope with stressful transitions ( Nielsen and Eaton, 1981; Lingard *et al.*, 2002; Meyer and Land, 2003; Manninen, Welin, Scheja and Silén, 2013). Mimicry does not include reflection, but if trickster-like experiences are not reflected upon in the learning process, students’ transition will not progress ( Turner, 1966; Davys and Beddoe, 2009). Consequently, the students have to actively enact the role of the portrayed video-case doctor to gain the insight. If not, the liminal phase is prolonged, whatever the good intentions of the video cases.

With a similar, but differently grounded, lens of social learning theory, the progression from novice to professional is described as a move from “legitimate peripheral participation” involving observation and imitation to “full participation” ( Lave and Wenger, 1991). From the liminal perspective, some students stayed positioned as “observers and imitators” in passive roles. This is important, since students having negative experiences related to professional socialisation processes in clerkship experience a major gap between preclinical and clinical training, regardless of the quality in the preclinical training ( Godefrooij, Diemers and Scherpbier, 2010; Petkari, Gutiérrez, Xavier and Küstner, 2018). Insufficient transition can ultimately prevent students from gaining the necessary professional understanding of future patient care situations ( Jones and Shapiro, 2012).

### The essential role of the ritual elder

The senior lecturer acted a guiding role of the liminal “ritual elder” to challenge old beliefs and bring new insights by subjecting students to the video cases as tricksters as intended. However, confronted with the additional unforeseen variety of trickster-like experiences in clerkship, students revealed a difference in how they navigated according to their perception of having support and encouragement in the clerkship.

The liminal construct considers support from a ritual elder significant in how participants undergoing transition may or may not navigate emotional disruptive learning situations to gain professional insight ( Turner, 1967; Meyer and Land, 2005). Lack of support may compromise the ability to redeem from disruptive trickster-like experiences encountered in practice ( Davis and Weeden, 2009; Szakolczai, 2015). The present study is in line with this conclusion as students who lacked guidance and support demonstrated problems with how to overcome emotive stress in their psychiatric clerkship and apply the insight from the video cases to their practice as intended. Their transition may thus be prolonged, as these students get ‘stuck’ in the liminal space, unredeemed from their disruptive reactions and emotions ( Meyer and Land, 2005; Davis and Weeden, 2009; Szakolczai, 2015). Lack of support and encouragement from a ritual elder in the threshold to and integration with clerkships may undermine preparatory teaching efforts to stimulate professional transition and patient-centred insight in students.

In conclusion, students, having support and encouragement in their psychiatric clerkship to explore patient perspectives, indicated patient-centred communication approaches when referring to how they had used insight from the preparatory video cases.

### Strengths and limitations

The rich picture interview approach in combination with the lens of liminal theory was useful in identifying and discussing transition in relation to emotions, perceptions, support, and experiences in the medical students’ perspective. While the use of individual rich picture interviews was effective in producing an in-depth picture, it might also explain the accentuated points of some students experiencing their psychiatric clerkship as negative or less optimal. Drawing attention to the students’ unredeemed experiences of chaos and uncertainty, characterising the state of liminality, the interview format could possibly serve as a safe-room of confession for suppressed and non-verbalised frustration. The methodology draws attention to students’ subjective emotional aspects limited to the video cases and diagnostic interview situations, whilst ignoring important contextual aspects such as organisational or cultural practices of which students may be unaware. However, the focus of this study was limited to the influence of video cases on subsequent clinical patient encounters. As the interview question directed the connection between video cases and students’ clerkship experience, we do not know if these reflections would have emerged on their own.

We acknowledge the limitations of our sampling and data collection. We used a convenience sample of students; nonetheless, those who volunteered were engaged and motivated to participate in the interviews. Although our data from 8 students have sufficient depth, results from a single institution may have limited transferability. Transferability of our qualitative findings is indecisive because interview questions following a semi-structured interview are not asked in a standardized way in all participants ( Malterud, 2001). Thus, regarding external validity, we do not know to what extent the study hypothesis or results apply to other settings. The rich description of our context and data allows the reader to decide to what extent our results could be transferred.

## Conclusion

The liminal perspective recognises medical students’ emotional experiences as powerful learning potentials, when confronted with the trickster-like abilities of video cases and patient encounters. With the rapid development of educational technologies in medical education, various blended learning formats such as self-directed learning, flipped classroom models, and long-distance, online learning emerge. The virtual and technology-based learning settings dissolve traditional liminal structures, including a physical teaching setting, a community of students, and a teacher in the role of a ritual elder, and call for attention to how learning experiences, professional transition and insight unfold in the expanding technological paradigm of medical education. Exploring how classical liminal structures unfold in virtual settings and blended learning formats could add to the understanding of how learning experiences gained with various educational technology influences students’ integration with their clinical practices.

## Take Home Messages

• Student engagement in clerkship learning seems highly influential on whether students adopt a patient-centered or self-centered focus from video cases.
• Lack of support and encouragement from a liminal ritual elder in the threshold to and integration with clerkships may undermine efforts to stimulate patient-centered awareness initiated with video cases in preparatory teaching formats.

## Notes On Contributors

**Kamilla Pedersen**, is an Assistant Professor at the Centre for Health Sciences Education, Aarhus University, Denmark with an interest in medical education. She holds a MA in educational anthropology and a PhD in Health Science.

**Dr. Anne Mette Moercke**, PhD is an associate professor at Copenhagen Academy for Medical Education and Simulation, Capital Region of Denmark.

**Dr Charlotte Paltved** is Clinical Associate Professor at Corporate HR, MidtSim, Central Denmark Region, Denmark.

**Dr Ole Mors**, PhD, is a Professor and Chair at Psychosis Research Unit, Aarhus University Hospital Psychiatry.

**Dr Charlotte Ringsted**, MD, MHPI, PhD, is Professor at Centre for Health Sciences Education, Faculty of Health, Aarhus University.

## Declarations

The author has declared that there are no conflicts of interest.

## Ethics Statement

The Danish Research Ethics Committee exempts studies of this type from review. Students who volunteered to participate in the interviews were informed of the intention of the study and how the data would be anonymised. The students signed a written consent form with the option to withdraw from the study at any time. The collected data were stored and anonymised in accordance with the guidelines from the Danish Data Protection Agency.

## External Funding

This study was in part funded by the Aarhus University, the Central Region of Denmark, and the Advisory Group for Medical Education North, Denmark.
