# Supplementary material for: Video cases as tricksters, in medical students´ transition to psychiatric clerkship. A liminal perspective
Source: MedEdPublish (2016). 2021 Apr 20;10:95. [Version 1] doi: 10.15694/mep.2021.000095.1 (PMC10939659; doi:10.15694/mep.2021.000095.1)
Supplement: Peer review history

# Reviewer report: Reviewer response for version 1

**DOI:** 10.21956/mep.18873.r26928

**Published:** 2021-10-13

**Authors:** Ken Masters

**Affiliations:** 1 Sultan Qaboos University

This review has been migrated. The reviewer awarded 4 stars out of 5 An interesting paper describing research grounded in liminal theory, using videos to assist medical students in their transition to psychiatric clerkship. Overall, the paper is well-written. As many readers may be unfamiliar with the theory (as it is drawn from anthropology), the authors have given a useful and succinct description of the pertinent theoretical aspects, in particular, the concept of the “trickster”; this is necessary for the reader to understand the rest of the paper.The research videos appear to have been well-constructed, and the interviews well-prepared. The results are interesting and useful, and have been handled sensitively by the researchers.There are a few issues with the paper, but these are mostly small, and do not detract from the overall value of the paper. • “The theoretical framework of this study is liminal theory (Adorno, Cronley and Smith, 2013).” I would not recommend using this reference as your citation for introducing the theory to the reader. When you first introduce a theory to the reader, it would be best to use the seminal texts in which the theory was first made public. The text that is supplied here (Adorno, G., Cronley, C. and Smith, K. S.) is not a seminal text on the theory, but is a research study that uses the theory. (It can be cited elsewhere, as an example of how the theory can be used in research, but the texts first used to introduce the theory to the reader should have been those by van Gennep and/or Turner (whom you do cite a few lines later).• To assist reproducibility, it would have been useful for the reader if the authors had given an indication of the resources (time/money/personnel) required to create the videos.• It is unfortunate that the authors have based their theming process on the work by Braun and Clarke, and then use the phrase “and themes emerged”, because it is specifically the work by Braun and Clarke that argues strongly against using this phrase (“themes do not just ‘emerge’.”). Perhaps the authors could have used more active terminology, as suggested by Braun and Clarke. • More details (especially demographics) of the students should have been given, and then indicated when reporting the quoted results.Overall, though, a well-constructed and useful paper.

Reviewer Expertise:

NA

No decision status is available

# Reviewer report: Reviewer response for version 1

**DOI:** 10.21956/mep.18873.r26929

**Published:** 2021-05-29

**Authors:** Megan Anakin

**Affiliations:** 1 University of Otago

This review has been migrated. The reviewer awarded 5 stars out of 5 I agree with the review comments from John Sandars, this is a strong article and its use to theory to frame the study offers helpful insights to medical educators. I recommend other to read it as well.To enhance this article, please consider the following suggestions.In the methods section, please explain more about the perspectives, assumptions, and expertise the three researchers brought to the study and the data analysis since it requires the reflexivity of the team to be exposed in order to appreciate the robustness, trustworthiness, and representativeness of the results. In the results section, please state how many students participated in the interviews and describe the demographics of the participants so the transparency, credibility and representativeness of the findings are clearer. I may have missed the reference to the number of participant and only found it in the last paragraph of the discussion section. In the results section, please consider providing the participants pseudonyms or random alphanumerical identity codes (such as P1, P2, …) so the reader can see that the quotations do not all come from one student. Right now, there is no way to see if the voice of more than participant is presented in the results or not other than assuming that the difference in drawing styles and number of drawings presented denotes different participants. The discussion section makes thoughtful connections to the literature and the strengths and limitations of the study are presented effectively.I would be very happy to read and review a revised version of this article.

Reviewer Expertise:

NA

No decision status is available

# Reviewer report: Reviewer response for version 1

**DOI:** 10.21956/mep.18873.r26930

**Published:** 2021-05-20

**Authors:** John Sandars

**Affiliations:** 1 Edge Hill University

This review has been migrated. The reviewer awarded 5 stars out of 5 There is a clear and theory-driven description and analysis using a visual research method. The format of MedEd Publish easily provides an opportunity to show some of the images. I hope that medical education researchers will be encouraged to take up visual research methods. If you need more persuasion about the value and insights of creative research approaches then have a look at David Gauntlett's book:Gauntlett D. Creative explorations: New approaches to identities and audiences. Routledge; 2007 https://davidgauntlett.com/portfolio/creative-explorations/ The importance of obtaining the students' reflections and meaning-making of their visual images is an essential aspect of visual research methods, especially with the added theoretical interpretive framework. The book by John Prosser provides some useful information:Prosser J, editor. Image-based research: A sourcebook for qualitative researchers. Routledge; 2005 Many thanks for for sharing this innovative study and I encourage all medical education researchers to read this article.

Reviewer Expertise:

NA

No decision status is available
